# The characteristics of the whole pelvic morphology in slipped capital femoral epiphysis: A retrospective observational study

**DOI:** 10.1097/MD.0000000000019600

**Published:** 2020-04-03

**Authors:** Masanori Wako, Kensuke Koyama, Yoshihiro Takayama, Hirotaka Haro

**Affiliations:** Department of Orthopaedic Surgery, Faculty of Medicine, University of Yamanashi, Chuo-shi, Yamanashi, Japan.

**Keywords:** acetabular anteversion, acetabular coverage, pelvic inclination, pelvic rotation, slipped capital femoral epiphysis

## Abstract

Slipped capital femoral epiphysis (SCFE) is a very common disorder affecting the adolescent hip. The etiology of SCFE is multifactorial and mechanical force associated with the characteristic morphology of the hip is considered one of the causes of SCFE. We investigated the characteristics of whole pelvic morphology including pelvic incidence (PI) in patients with SCFE and compared it with pelvic morphology in healthy children. We retrospectively assessed the whole pelvic morphology of 17 patients with SCFE and 51 healthy children using their pelvic computed tomography data. We measured superior iliac angle, inferior iliac angle, and ischiopubic angle as the parameters of pelvic rotation. Additionally, we measured acetabular anteversion of the superior acetabulum (AVsup) and of the center of the acetabulum (AVcen), and measured anterior acetabular sector angle (AASA), posterior acetabular sector angle, and the superior acetabular sector angle (SASA) as parameters of acetabular coverage and PI. Each measurement was compared between the 2 groups. AASA and SASA of patients with SCFE were significantly greater than that of controls, and AVsup of patients with SCFE was significantly smaller. There were no significant differences in pelvic rotation, PI, or AVcen between the 2 groups. This is the 1st report to evaluate SCFE patients’ whole pelvic morphology including PI and pelvic rotation. Our results showed that patients with SCFE have excessive coverage of the anterior and superior acetabulum, and a more retroverted cranial acetabulum as compared with healthy control subjects. Such characteristic pelvic morphology may be involved in the onset of SCFE. To clarify the mechanical forces involved in SCFE onset, further investigations of pelvic morphology and alignment, including the femur and spine, are needed.

## 1 Introduction

Slipped capital femoral epiphysis (SCFE) is one of the most common disorders affecting the adolescent hip. The etiology of SCFE is multifactorial, involving several biochemical aspects and mechanical factors that include obesity, acetabular retroversion,^[1–3]^ the capital femoral physeal slope,^[4–6]^ femoral retroversion,^[7]^ and the size of the epiphyseal tubercle.^[8]^ These anatomical abnormalities may generate abnormal biomechanical forces at the physis.

There have been several studies citing acetabular version as a cause of SCFE, but controversy still exists. Recently, Gebhart et al reported that specimens with SCFE deformity demonstrated a smaller pelvic incidence (PI) than a large cohort of normal control subjects.^[9]^ PI is the angle between the line perpendicular to the sacral plate at its midpoint and the line connecting this point to the axis of the femoral heads. It is a position-independent anatomic parameter that is one of several factors to determine lumbar lordosis and pelvic orientation. PI has been studied extensively in relation to spine pathology; numerous studies have shown that increased PI transmits greater mechanical force to the lumbar spine. Pelvic tilt affects the shear force applied to the hip joint, so PI may be implicated in the etiology of SCFE. However, there has been no report to follow or expand upon Gebhart's study. If the SCFE patient's whole pelvic morphology including PI, which is one of the factors that defines pelvic inclination was clarified, it will be very useful information for establishing the treatment method of SCFE and elucidating the causes of SCFE.

The purpose of this study is to investigate the whole pelvic morphology including PI of patients with SCFE using computed tomography (CT) and to compare it with morphology in healthy children.

## 2 Materials and methods

We identified 17 patients with SCFE who underwent pelvic CT between 2011 and 2018. Two patients had bilateral SCFE and 15 patients had unilateral SCFE. All patients underwent CT imaging prior to surgery as part of treatment planning. In 7 of the 19 hips, the severity of SCFE was mild; in 6, moderate; and in 6, severe.^[10]^ Based on Lorder scale, 16 hips were stable and 3 hips were unstable.^[11]^

Control subjects were identified from the imaging database by a search of patients who underwent pelvic CT for several reasons between January 2009 and January 2017. Children aged 3 to 18 years old were included. We excluded patients with a history of symptoms about the hips, obvious abnormalities in skeletal development, or history of treatment affecting bone growth. Ninety-seven patients matched the criteria. From the 97 patients we extracted, 51 patients matched to the 17 patients with SCFE on the basis of gender and age and defined the 51 patients as healthy controls. In all patients, we measured and compared the pelvic rotation, acetabular coverage, acetabular version, and PI.

First, we reconstructed the data from each patient based on the tomographs in Digital Imaging and Communications in Medicine (National Electrical Manufacturers Association) with the processing and analysis software (SYNAPSE VINCENT; Fujifilm, Tokyo, Japan), and measured parameters that define pelvic morphology. To eliminate possible measurement errors, pelvic position was corrected digitally as follows. In the coronal plane, the pelvis was aligned horizontal to the line connecting the inferior aspects of the bilateral teardrops. In the axial plane, the pelvis was aligned vertical to the line connecting the pubic symphysis and the center of the sacrum. PI in the sagittal plane was aligned with the line connecting the bilateral anterior superior iliac spine (ASIS) and the pubic tubercle.

**Figure 1:**
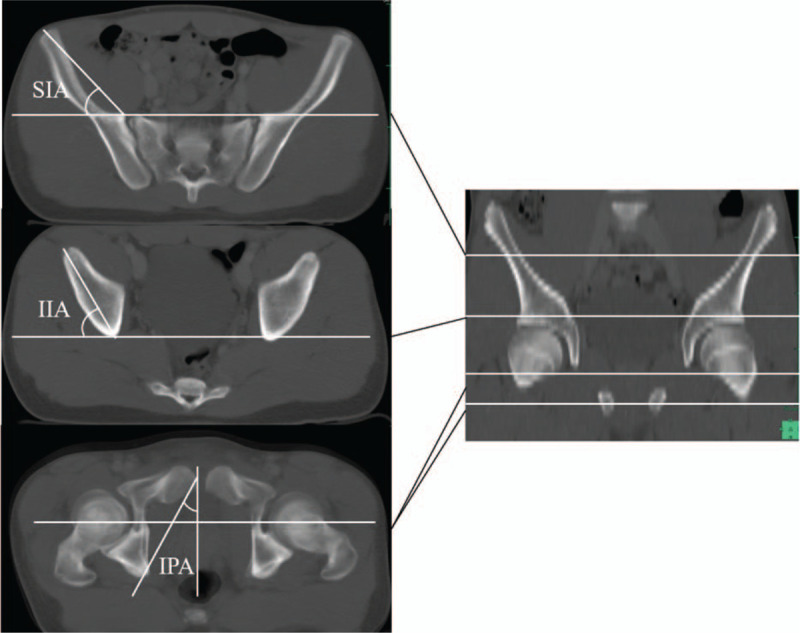
We adopted the superior iliac angle (SIA), inferior iliac angle (IIA), and ischiopubic angle (IPA) as the parameters indicating the pelvic winging. SIA is formed by the intersection of a line connecting the medial edge of the anterior superior iliac spine and the anterior margin of the sacroiliac joint, and a horizontal line on the axial plane. IIA is formed by a line connecting the anterior aspect of the anterior inferior iliac spine and the posterior aspect of the ilium, and a horizontal line on the axial plane. IPA is a projection angle formed by the intersection of a line connecting the anterosuperior edge of the pubic symphysis and the ischial spine and a sagittal line on the axial plane for which we superimposed the sections that passed through the ischial spine and the pubic symphysis.

As the parameters of rotational alignment of the innominate bone (pelvic rotation), we measured the superior iliac angle (SIA), inferior iliac angle (IIA), and ischiopubic angle (IPA), which were defined by Fujii et al.^[12]^ SIA is formed by the intersection of a line connecting the medial edge of the ASIS and the anterior margin of the sacroiliac joint, with a horizontal line in the axial plane. The IIA is formed by a line connecting the anterior aspect of the anterior inferior iliac spine and the posterior aspect of the ilium, with a horizontal line in the axial plane. IPA is a projection angle formed by the intersection of a line connecting the anterosuperior edge of the pubic symphysis and the ischial spine with a sagittal line in the axial plane on which we superimposed the sections that passed through the ischial spine and the pubic symphysis (Fig. 1).

**Figure 2:**
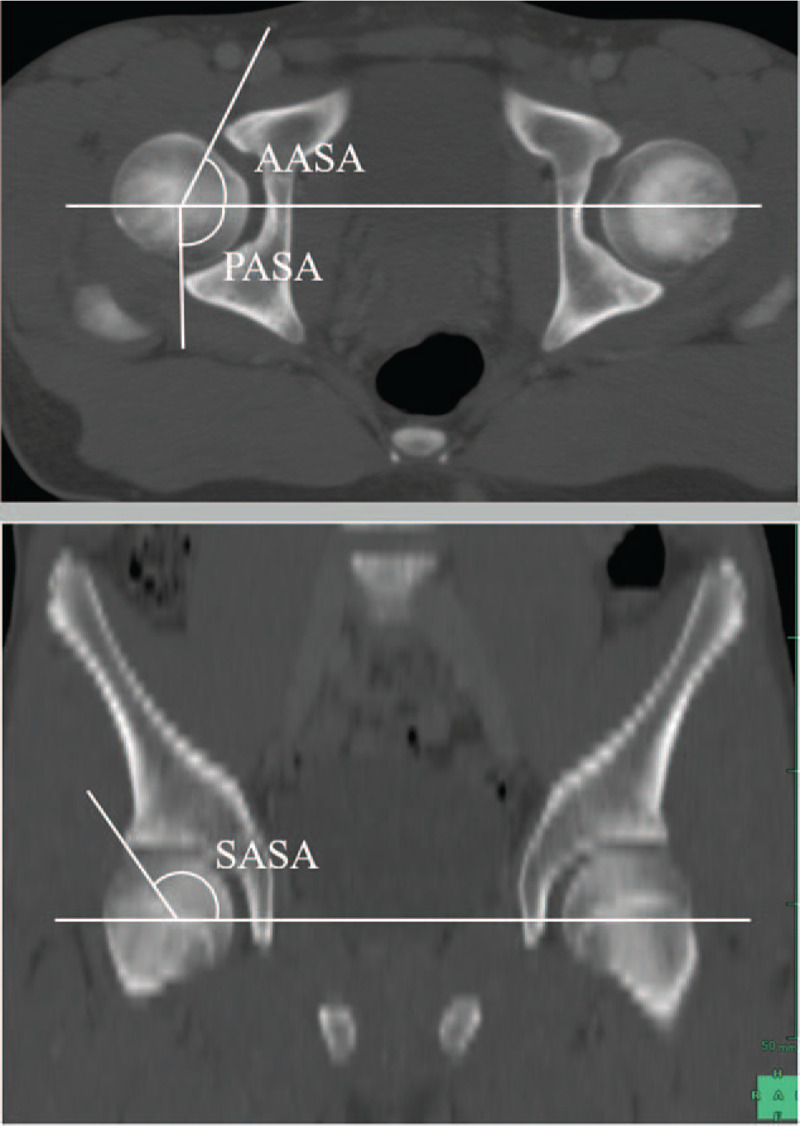
The acetabular sector angle is formed by the intersection of a line connecting the femoral head center and the acetabular edge. The acetabular sector angle was measured in anterior, superior, and posterior directions. AASA = anterior acetabular sector angle, PASA = posterior acetabular sector angle, SASA = superior acetabular sector angle.

To assess the parameters of acetabular coverage on the femoral head, we measured the anterior acetabular sector angle (AASA), posterior acetabular sector angle, and superior acetabular sector angle (SASA). According to the method described by Anda et al^[13]^ and Fujii et al,^[12]^ we used a horizontal line as the baseline of the measurement and determined the angle in anterior, superior, and posterior directions (Fig. 2).

**Figure 3:**
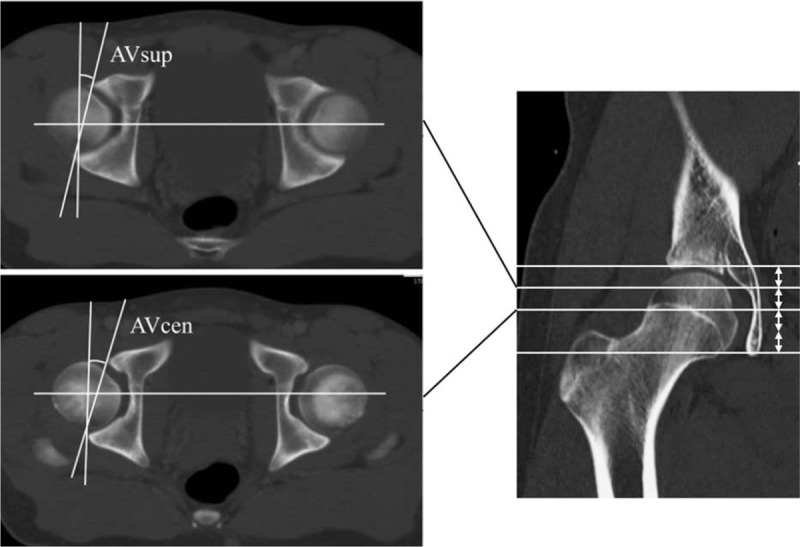
Acetabular anteversion of the superior acetabulum (AVsup) is measured on the axial plane corresponding to the proximal 1/4 of the distance between the acetabular roof and the inferior pelvic teardrop on the coronal plane. Acetabular anteversion of the center of the acetabulum (AVcen) is measured on the axial plane corresponding to the center of the femoral head on the coronal plane.

We measured acetabular version using 2 different measurement parameters. Acetabular anteversion of the superior acetabulum (AVsup) was defined as the acetabular version on the axial slice corresponding to the proximal 1/4 of the line joining the acetabular roof to the inferior pelvic teardrop, as described by Monazzam et al.^[3]^ AVcen was defined as the acetabular version on the axial slice of the acetabulum corresponding to the center of the femoral head (Fig. 3).

**Figure 4:**
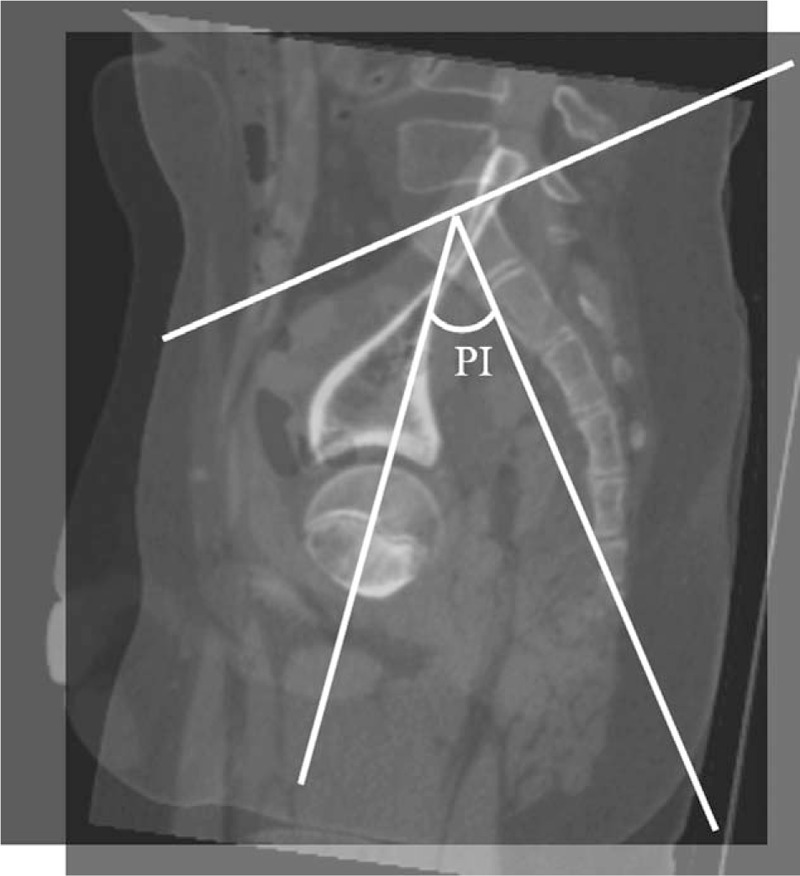
Pelvic incidence (PI) measurement as shown from the sagittal planes of computed tomography.

For PI, we measured the angle between the line perpendicular to the sacral plate at its midpoint and the line connecting this point to the axis of the femoral heads in the sagittal plane passing through the center of the sacrum, and the sagittal plane passing through either femoral head (Fig. 4).

Statistical analysis was performed using SPSS version 25.0. In both control and SCFE groups, the extent of correlation of the left and right measurements without PI was highly significant (Pearson correlation coefficient), so the mean of the left and right angles was taken as a single measure for this study (Table 1). Each measurement was compared between groups using the Mann–Whitney *U* test, and the significance was set at *P* < .05. To evaluate intraobserver agreement, all measurements for 20 randomly selected control cases were repeated by the same reader (MW) during the course of 2 sessions at least 1 month apart. For interobserver agreement, a 2nd reader (YT) repeated the measurements for the same 20 patients. Interobserver and intraobserver reliabilities for pelvic measurements were assessed by estimating intraclass correlation coefficients (ICCs) along with 95% confidence intervals using an ICC (2,1) modeling scheme. Intraobserver and interobserver correlations indicated substantial agreement (ICC, >0.7) for all measurements (Table 2).

**Table 1:**
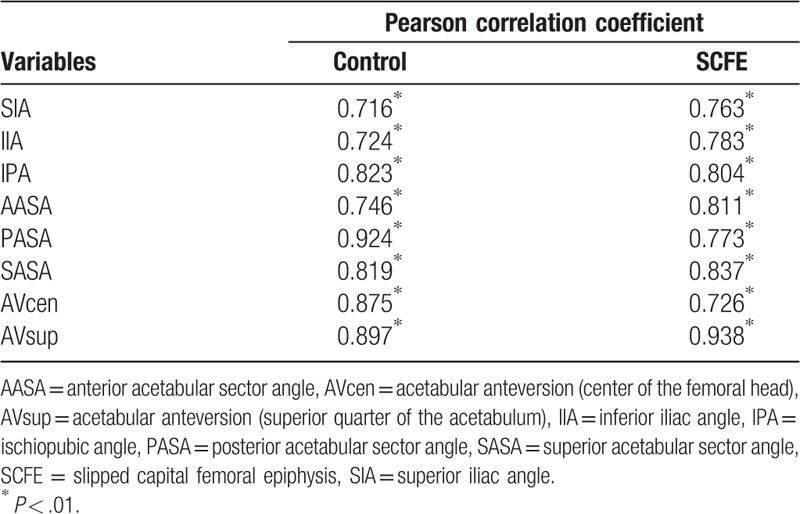
Correlation of left and right pelvic morphology in both groups.

**Table 2:**
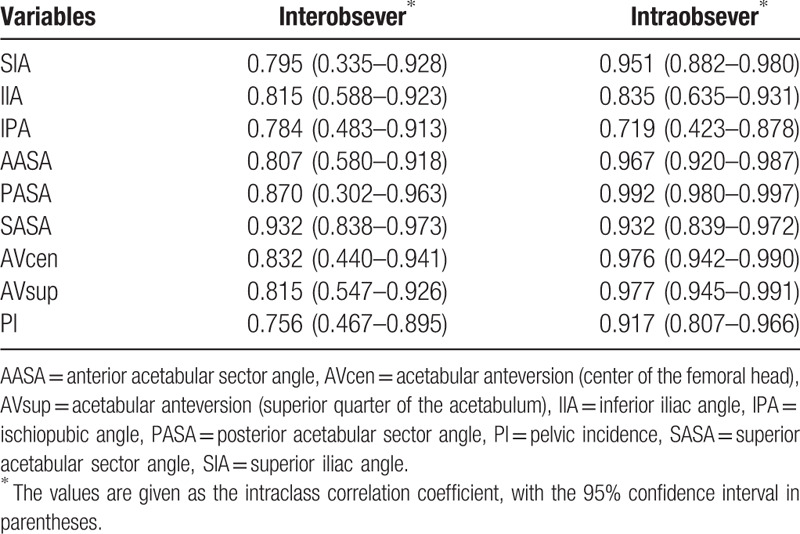
Reliability of each measurement (N = 20).

This retrospective study was approved by the institutional review board of our university (University of Yamanashi Faculty of Medicine Ethics Committee: No. 1106) on September 11, 2013.

## 3 Results

The mean age, gender distribution, and each measurement of both groups are shown in Table 3. As groups were matched, there was no difference in mean age or gender distribution between the 2 groups.

**Table 3:**
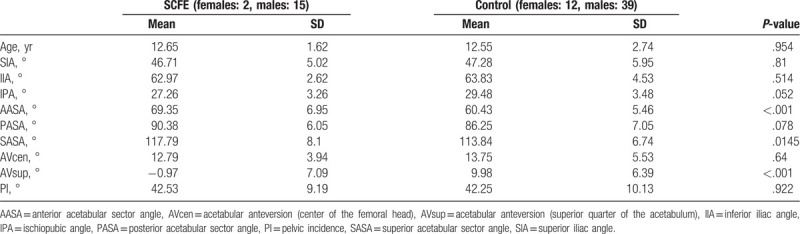
Results of all morphologic variables and age.

### 3.1 Pelvic rotation

There was a tendency for the IPA of the SCFE group to be smaller than the control group, but the groups were not significantly different. Also, there were no significant differences between the SCFE group and the control group for SIA or IIA.

### 3.2 Acetabular coverage

The AASA and SASA of the SCFE group were significantly greater than for the control group. The differences between groups for the mean AASA were particularly large.

### 3.3 Acetabular version

AVcen was not significantly different between the SCFE and control groups, but the mean AVsup of the SCFE group was <0 and was significantly smaller than that of the control group.

### 3.4 Pelvic inclination

There was no significant difference between the 2 groups for PI.

## 4 Discussion

We investigated the whole pelvic morphology of patients with SCFE using CT scans and compared it with the morphology in healthy children. This is the 1st report to evaluate SCFE patients’ whole pelvic morphology including PI or pelvic rotation.

In this study, we found that AASA and SASA of the patients with SCFE were significantly greater than for control subjects, and AVsup of the patients with SCFE was significantly smaller than in control subjects. There were no significant differences in pelvic rotation, PI, or AVcen between the 2 groups.

As for pelvic rotation, Fujii et al reported that the internal rotation of the innominate bone in patients with developmental dysplasia of the hip was greater than in control patients. This internal rotation was associated with increased acetabular anteversion angle and acetabular inclination angle.^[12]^ However, to the best of our knowledge, there has been no report on the pelvic rotation of patients with SCFE, and the results of the present study suggest that pelvic rotation is not related to the onset of SCFE. Our results were different from Fujii's report in that there was no correlation between the pelvic rotation and the acetabular version.

There have been some reports about acetabular coverage in patients with SCFE. Monazzam et al reported that superior acetabular coverage (lateral center edge angle) of SCFE hips was significantly larger than in control hips,^[3]^ and Sankar et al reported the overcoverage of the superior acetabulum in patients with SCFE.^[2]^ Our results support these reports, and these results of large AASA and SASA seem almost equivalent to the cranial acetabular retroversion described as follows.

There is still controversy about acetabular version in patients with SCFE. There are many reports indicating that there is no difference between the acetabular version of patients with SCFE and healthy control patients^[9,14–16]^; however, many reports also indicate that patients with SCFE have a tendency for acetabular retroversion.^[1–3]^ But in all of the reports showing no difference, acetabular version was measured in the axial plane passing through the center of femoral head. On the contrary, most reports that measure the acetabular version of the proximal portion of the acetabulum support the conclusion that patients with SCFE exhibit retroversion, and the same result was obtained in this study. We conclude that although the anteversion at the center of the hip varies widely, there is no doubt about the tendency for cranial acetabular retroversion in patients with SCFE.

If the morphology of the acetabulum is mechanically related to the onset of SCFE, the morphology of the cranial portion of the acetabulum is more critical than the center of the acetabulum because body weight is applied to the acetabular roof. Therefore, we believe that the onset of SCFE is determined by retroversion or coverage at the cranial acetabulum, not the center of the acetabulum. For that reason, we suggest that the cranial acetabular retroversion shown in this study is critical for the onset of SCFE.

There is only 1 report that mentions the PI of patients with SCFE, and this report states that PI is significantly smaller in patients with SCFE.^[9]^ Conversely, there was no significant difference in PI between the 2 groups in the present study. PI was introduced by Duval-Beaupère et al as a means to evaluate sagittal balance of the spino-pelvic-hip complex,^[17]^ and a number of studies have stated the relationship between PI and several spinal diseases.^[18–21]^ The PI is regarded as an important parameter when studying not only spinal disorders but also hip diseases.^[22,23]^ Boulay et al described the PI as an important biomechanical tool around the hip to determine the range of pelvic sagittal motion specific for an individual.^[23]^ The femoral head and the acetabulum have a reciprocal interaction in which proximal femoral morphology can compensate for acetabular morphology and vice versa.^[24–26]^ Gebhart et al stated that the disruption of the spinal-pelvic alignment often results in a displacement of load absorption. The transfer of displaced mechanical forces may increase the load on vertebral endplates, their associated disks, and the femoro-acetabular joints.^[9]^ Although the results of the present study are different from those of Gebhart's report, we agree that spino-pelvic alignment may be important for the onset of SCFE. When considering the load on the hip joint, the morphology and alignment of the pelvis and spine in a standing position should be assessed in combination. As a future plan, we would like to evaluate the pelvic morphology, femoral morphology, and PI of patients with SCFE together.

The weakness of this study is the small number of patients. Although there are clear statistical differences between groups in this study, a future task is to increase the number of patients and to extend and validate these results. In addition, we cannot completely rule out the possibility that a case included in the control group will suffer from SCFE in the future. If so, the case is not appropriate as a control case. However, so far, no case in control group that subsequently developed SCFE has been identified.

## 5 Conclusion

This is the 1st report of whole pelvic morphology including PI or pelvic rotation in patients with SCFE compared to a matched control group. Our results show that patients with SCFE have excessive coverage of the anterior and superior acetabulum, and a more retroverted cranial acetabulum, when compared with controls. Such characteristic pelvic morphology may be part of the pathogenesis for the onset of SCFE. To clarify the mechanical forces involved in onset of SCFE, further investigation of pelvic morphology and alignment including the femur or spine is needed.

## Acknowledgment

The authors thank JAM Post for help with language editing of the manuscript.

## Author Contribution

**Conceptualization:** Masanori Wako, Kensuke Koyama.

**Data curation:** Masanori Wako, Yoshihiro Takayama,

**Formal analysis:** Masanori Wako.

**Investigation:** Masanori Wako.

**Writing – original draft:** Masanori Wako.

**Writing – review & editing:** Masanori Wako, Hirotaka Haro.
